# Innate Immunity at the Core of Sex Differences in Osteoarthritic Pain?

**DOI:** 10.3389/fphar.2022.881500

**Published:** 2022-05-19

**Authors:** Natália Valdrighi, Juliana P. Vago, Arjen B. Blom, Fons A.J. van de Loo, Esmeralda N. Blaney Davidson

**Affiliations:** Experimental Rheumatology, Radboud University Medical Center, Nijmegen, Netherlands

**Keywords:** innate immunity, osteoarthritis, sex differences, pain, inflammation

## Abstract

Osteoarthritis (OA) is a progressive whole-joint disease; no disease-modifying drugs are currently available to stop or slow its process. Symptoms alleviation is the only treatment option. OA is the major cause of chronic pain in adults, with pain being the main symptom driving patients to seek medical help. OA pathophysiology is closely associated with the innate immune system, which is also closely linked to pain mediators leading to joint pain. Pain research has shown sex differences in the biology of pain, including sexually dimorphic responses from key cell types in the innate immune system. Not only is OA more prevalent in women than in men, but women patients also show worse OA outcomes, partially due to experiencing more pain symptoms despite having similar levels of structural damage. The cause of sex differences in OA and OA pain is poorly understood. This review provides an overview of the involvement of innate immunity in OA pain in joints and in the dorsal root ganglion. We summarize the emerging evidence of sex differences regarding innate immunity in OA pain. Our main goal with this review was to provide a scientific foundation for future research leading to alternative pain relief therapies targeting innate immunity that consider sex differences. This will ultimately lead to a more effective treatment of pain in both women and men.

## Introduction

Osteoarthritis (OA) is a musculoskeletal disease and the most common form of arthritis, affecting more than 300 million people worldwide. Age is an important risk factor for OA and is the leading cause of disability among older adults over 60 years old (Murray et al., 2012; Hunter et al., 2020). As OA incidence is constantly increasing in the elderly population, these numbers are expected to increase further. OA is a highly heterogeneous condition and can affect different joints: knees, hips, hands, spine, and feet, among others. Hand and knee OA are the most common forms (Haugen et al., 2011; Callahan et al., 2015). Common symptoms are joint pain, stiffness, and decreased functional mobility, with joint pain being the main reason driving OA patients to seek medical help. OA is a progressive whole-joint disease and no disease-modifying drugs are currently available to stop or slow its process, resulting in symptom alleviation as the main treatment option. Upon knee and hip joint failure, total joint replacement is often the last medical solution, but unfortunately does not alleviate pain in all patients (Lundblad et al., 2008; Wylde et al., 2017; Wylde et al., 2018). For all other joints, this is either impossible or not a common solution. The importance of pain as a burden, along with this pain being persistent, places OA as a major cause of chronic pain in adults and the fourth cause of disability worldwide (Murray et al., 2012).

Pain, including acute pain, is a complex phenomenon that evolved as a protective action in response to a perceived threat, associated with actual or risk of tissue damage. Pain is projected in the conscious domain by the brain, resulting from a series of complex interactions within multiple systems in the body (Raja et al., 2020). Mechanistically, a noxious (pain) signal starts in the periphery (e.g., OA joint) with the detection and transmission of the noxious stimulus by activated primary afferents (neurons). The action potential (the noxious stimuli converted to an electric signal by the neurons) is then transmitted to the dorsal root ganglion (DRG) where the cluster of cell bodies is located. From there, the painful signal is conveyed by extending axons from primary neurons to the dorsal horn in the spinal cord, which is then relayed via the lateral spinothalamic tract to higher central circuits in the brain. The pain signal is decoded in the somatosensory cortex, bringing pain into the conscious domain (Basbaum et al., 2009). Chronic pain has a complex and poorly understood pathogenesis, which was beyond scope of our review. In a minimalist summary, the input in the nociceptive system is amplified via peripheral (Apkarian and Reckziegel, 2019), spinal (Basbaum et al., 2009), or higher central circuit routes (Apkarian et al., 2011), which eventually create and maintain a chronic pain brain state even after the noxious stimulus has been cleared (Apkarian et al., 2005).

**FIGURE 1:**
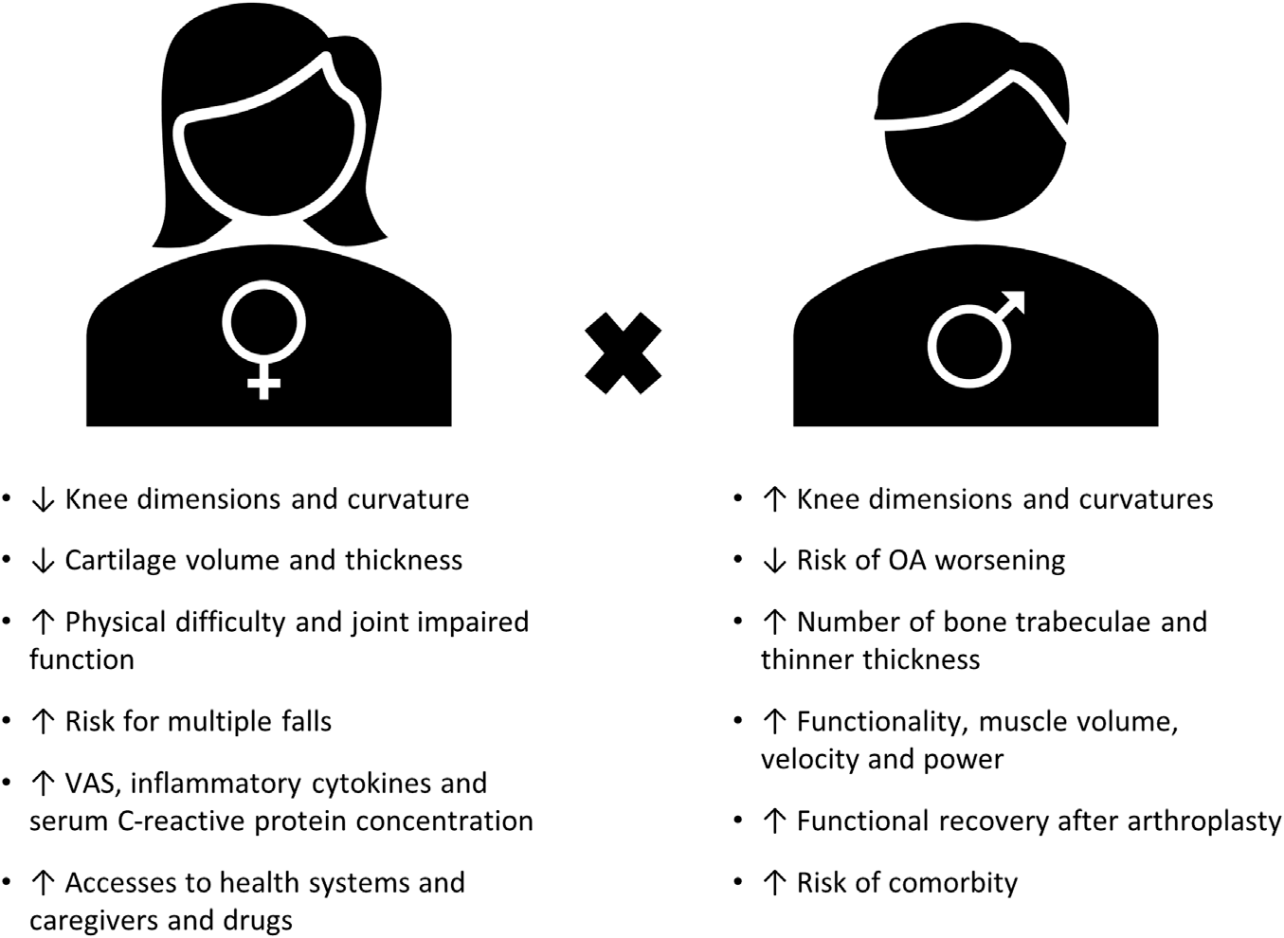
Summary of major clinical findings of sex differences in OA (adapted from (Basbaum et al., 2009; Tschon et al., 2021)).

Remarkably, sex is one of the predisposing risk factors of chronic pain, with the majority of patients with chronic pain being women (Ruau et al., 2012). In agreement, OA also affects a higher proportion of women than men (Srikanth et al., 2005; Glass et al., 2014). Increasing numbers of researchers have focused on understanding sex differences in OA in the past decade from both the preclinical (for a systematic review, see (Contartese et al., 2020)) and clinical (for systematic review, see (Tschon et al., 2021)) perspectives. Even though hand OA is more prevalent in women (Haugen et al., 2011), clinically, knee and hip OA have been the most investigated for sex differences (Tschon et al., 2021). Therefore, we focused on these joints in this review, unless otherwise specified. The major clinical findings between sexes are summarized in Figure 1, including anatomic differences regarding morphometry and kinetics between men and women that now are being quantified and described. These differences might shed light on the potential effect of sex on joint loading, OA severity, and poorer outcome after total knee arthroplasty (Berger et al., 2012; Callahan et al., 2015; Gustavson et al., 2016; Bigham et al., 2018). Differences in pain experience between sexes have also been reported in OA, with women reporting more knee pain (by visual analog scale (VAS)) than men (Glass et al., 2014), regardless of Kellgren–Lawrence grade (Cho et al., 2010). However, the biomechanical component alone does not explain the sex differences in OA pain observed in clinical practice. Women reporting worse pain scenario (Perruccio et al., 2017), increased VAS score (Glass et al., 2014; Solheim et al., 2017), and lower pain threshold (Pan et al., 2016) have been linked to an enhanced inflammatory response. Yet, despite the increase in researchers investigating these sex differences, the biological mechanisms behind these differences in OA pain appear to be understudied (Tschon et al., 2021).

To add to the complexity, growing amounts of evidence in pain research support that pain pathways are remarkably different between the sexes (for a review, see (Sorge and Totsch, 2017)). This body of evidence suggests that sex differences in pain involve differences in key cells of the innate immunity: the macrophages and the macrophage-like microglia in the pain pathway (Sorge et al., 2011; Sorge et al., 2015; Luo et al., 2021). The innate immune system is not only linked to OA pathophysiology (for a review, see (Barreto et al., 2020)), but also to OA pain mechanisms (for a review, see (Miller R. J. et al., 2020)). The majority of the scientific knowledge (including OA research) developed so far has largely overlooked sex as a potential biological variable. This is reflected in the majority of analgesics on the market, which were mostly developed based on research on men only. As sex differences in biology of pain are now becoming more evident, they may explain why women with OA are at a higher risk of receiving inadequate pain relief (Conaghan et al., 2015).

With this review, we aimed to provide an overview of sex-inclusive OA research on peripheral pain, focused only on the joint and DRG compartments, and how they intertwine with innate immunity at the core of sex differences in OA pain. Our final goal with this review was to provide a scientific foundation for future research leading to new pain relief therapies that consider the differences between sexes.

## Important Disease Mechanisms in OA Pain

In OA, pain initially has a strong biomechanical component. Abnormal loading from the damaged joint activates mechanoreceptors, generating pain characterized as nociceptive pain (Heppelmann and McDougall, 2005). This is in line with what patients describe during early OA: pain exacerbation during movement and relief during rest (Hunter et al., 2008; Neogi, 2013). As the disease advances, inappropriate joint stress can lead to more damage to the cartilage extracellular matrix (ECM), exposing the nociceptor nerve endings in the subchondral bone to the intra-articular space. Products from the breakdown of this tissue promote the activation of innate immunity, releasing a cocktail of inflammatory and pronociceptive mediators in the intra-articular space (Maldonado and Nam, 2013; Liu-Bryan and Terkeltaub, 2015). In patients, OA progression is associated with low-grade inflammation in the synovium (synovitis) in at least 50% of the cases (Ayral et al., 2005; D’Agostino et al., 2005). Synovitis is a product of the activity of the innate immune system trying to restore tissue homeostasis upon tissue damage (Orlowsky and Kraus, 2015). The consensus is that synovitis is the only OA feature that has been clinically associated with pain sensitization (Neogi, 2013; Neogi et al., 2016). The proalgesic cocktail then promotes activation of the nerve endings in the synovium and the menisci, and then exposes nerve endings in the bone. Nociceptor activation results in peripheral sensitization, characterized as inflammatory pain. As this process persists and the primary afferents are constantly in an activated state, excitability changes are promoted in the secondary neurons in the dorsal horn of the spinal cord, making them hyperreactive, resulting in central sensitization (Schaible et al., 2009). A subgroup of OA patients (around 30%) show signs of hyperexcitability of the central nervous system (CNS), characterized by widespread hyperalgesia and allodynia, and abnormal spatial and temporal summation, among others (Lluch et al., 2014). Despite OA pain being classically accepted as nociceptive and more recently gaining prominence as inflammatory pain, some findings have suggested that nerve damage in the joint, DRG, and spinal cord may also occur in some cases (Ivanavicius et al., 2007; Orita et al., 2011a; Thakur et al., 2012; Dimitroulas et al., 2014; McDougall et al., 2017; McDougall and O'Brien, 2020). Patients at a later OA stage describe the pain as either intermittent but generally severe/intense or as persistent background pain/aching, which is a characteristic pain pattern for nerve damage (Hunter et al., 2008; Neogi, 2013). Additionally, central sensitization and possible neuropathic mechanisms may contribute to the pain after total knee arthroplasty (Wylde et al., 2018). Peripheral input in the joint is responsible for starting the broad range of pain symptoms in OA patients. This input is closely linked to the innate immunity activity; hence, targeting its response may prevent worsening of the pain scenario.

## Innate-Immunity-Related OA Pain

The origin of OA origin is multifactorial, in which biomechanical, genetic, systemic, and environmental factors play a role in OA development (Busija et al., 2010). More recently, inflammation was found to play a key role in the pathogenesis of OA, with the innate immune system being one of its main contributors (Liu-Bryan, 2013). Synovitis is considered a secondary process in the joint following cartilage injury, inducing innate immune activation, which provides a critical link in the initiation and progression of OA (Scanzello and Goldring, 2012). Changes in the osteoarthritic joint include cartilage erosion, chondrocyte hypertrophy, and the production of matrix degradation products. In turn, these alterations lead to synoviocyte (synovial fibroblast) proliferation; the infiltration of macrophages, mast cells, and lymphocytes; and the release of proinflammatory mediators (Goldring et al., 2008). As this process evolves, changes occur in the subchondral bone: sclerotic bone, osteophyte formation, and subchondral bone marrow lesions with altered vascular and neuronal innervation. During the early stages of OA, tissue injuries accumulated over time may lead to the release of damage-associated molecular patterns (DAMPs), which are recognized by pattern recognition receptors (PRRs), such as Toll-like receptors (TLRs) and NOD-like receptors (NLRs) (Chen and Nunez, 2010). The activation of PRRs may, in turn, trigger a local innate immune system reaction (Liu-Bryan and Terkeltaub, 2015). Consequently, inflammatory pathways may be activated in resident cells, leading to upregulation of several inflammatory mediators and cartilage matrix degrading proteases (Maldonado and Nam, 2013).

The production of proinflammatory mediators as well as cell–cell interactions in the joint have been suggested to be involved in OA pain, indicating the innate immune system as a key player in the molecular mechanisms of OA pain (for a review, see (Miller R. J. et al., 2020)). For a long time, pain was acknowledged as solely a nervous system process, with immune cells participating in the periphery, at the initiation of the pain signal. However, despite the nervous and the immune systems being two distinctly complex systems, they share similar protective functions: host defence and survival (Chiu et al., 2012; Pinho-Ribeiro et al., 2017; Baral et al., 2019). These systems are more intertwined than researchers previously thought. Neurons express multiple shared receptors with the immune system, including the aforementioned PRRs: PAMPs and DAMPs, such as the TLR family and chemokine and cytokine receptors. These two systems evolved with fast and efficient bidirectional communication, which could have occurred through multiple pathways. Neurons can be directly activated via the PRR, resulting in changes in excitability and the release of inflammatory mediators, including chemokines, cytokines, and neuropeptides, which promote and facilitate the inflammatory response. Indirect changes in nociceptor sensitivity can occur when immune cells signal through the production of pro- or antinociceptive molecules, which then leads to nociceptors modulating immune functions (Chiu et al., 2012; Pinho-Ribeiro et al., 2017; Baral et al., 2019). The immune system and the nervous system were more recently found to communicate at multiple sites along the pain pathway. These sites include the periphery, DRG, spine, and even the brain. This bidirectional crosstalk has been gaining increasing attention in the field of chronic pain (for reviews, see (Hore and Denk, 2019; Grace et al., 2021)), OA pain (for a review, see (Geraghty et al., 2021)), and sex differences in pain (for reviews, see (Rosen et al., 2017; Gregus et al., 2021)).

A growing body of evidence indicates that TLR4 plays a critical role in the induction and maintenance of pain (Bruno et al., 2018) and is particularly involved in the pathogenesis of OA (Scanzello et al., 2008). The recognition of DAMPs from damaged tissue by TLRs is a protective mechanism. However, continuous or excessive activation of TLRs leads to a sustained production of proinflammatory mediators. Despite the existence of other TLRs, TLR4 senses more DAMPs than any other known PRR, including those produced in the OA joint (Goldring and Scanzello, 2012; Gómez et al., 2015). TLR4 activation and then the consequent NF-κB activation induce the production of cytokines (e.g., IL-6, IL-1β, and TNF) and chemokines (e.g., IL-8, CCL2, and CCL5), which promote a proinflammatory milieu in the joints of OA patients, as well as leukocyte recruitment (Scanzello et al., 2008; Scanzello, 2017). TLR4 is constitutively expressed in the majority of leukocytes in synovial membranes during OA (Radstake et al., 2004) and is upregulated in lesioned cartilage areas (Kim et al., 2006). TLR4 can excite DRGs in a murine model of OA (Miller et al., 2015). Additionally, TLR4 is present in a soluble form in the synovial fluid of OA patients, and was associated with OA severity, suggesting TLR4 as a potential OA biomarker (Barreto et al., 2017). The findings of preclinical studies have suggested that differences in innate immunity, associated with TLR4 activation, may contribute to sex differences in pain (Sorge et al., 2011; Sorge et al., 2015; Szabo-Pardi et al., 2021). In the next section, we describe how inflammatory mediators contribute to OA pain, highlighting recent findings showing differences between sexes.

## Sex Differences and Innate-Immunity-Related OA Pain Mediators

Little is known about the differences between the sexes in the interface between innate immunity and OA pain. Correlations between reported OA pain and inflammatory factors have been described in the synovial fluid, cerebrospinal fluid (Kosek et al., 2018), and serum (Perruccio et al., 2019) in clinical studies comparing women and men. A summary of the major findings is provided in Table 1. We discuss these and additional studies in the following sections. We discuss the members of innate immunity that play an important role in the mechanism of OA that have been linked to OA pain with a focus on sex differences.

**TABLE 1:** Main sex differences and OA association to innate immune system activation.

Preclinical Studies	Outcome	References
OA FLS (*in vitro*)	↑ iNOS, IL-1β, and CCL2 in females; ↑ macrophage attraction to OA FLS (TNF-stimulated) in females	Xue et al. (2018)
TMJOA model in rats	↑ OA severity in females; ↑ iNOS, IL-1β, CCL2, and CD68 in synovial membrane in females	Xue et al. (2018)
**Clinical studies**		
** **Knee OA – SF (10 men and 10 women)	↑ IL2α, IL3, IL12p40, IL16, and TNFβ in women; ↑ macrophage stimulators LIF, M-CSF, MIF in women; ↑ pro-inflammatory mediators GRO-α, MCP-3, MIG in women	Pan et al. (2016)
** **Knee OA – SF (21 men and 23 women)	↑ IL-8, CCL-4, and MCP-2 in men; ↑ Acute pain (VAS score) after knee arthroscopy in women compared with men	Solheim et al. (2017)
** **Knee OA – Serum, SF, CSF (23 men and 17 women)	IL8 (synovial fluid) positive correlation with VAS pain in women, but not men ↑ CCL2 levels significantly higher in CSF than serum, with positive correlations for CCL2 across CSF, serum and SF, in women but not in men	Kosek et al. (2018)
** **Knee OA – SF (24 men and 29 women)	↑ macrophages and ↓ monocytes in women	Kriegova et al. (2018)
** **Knee OA – Serum (81 men and 115 women)	↑ knee pain scores in women; ↑ TNF-α in men than women IL8 and IL-1β positive correlation with knee pain (WOMAC knee pain score) in men and negative correlation for women IL6 negative correlation with knee pain in men and positive correlation in women	Perruccio et al. (2019)
** **Knee OA- Serum (36 men and 48 women)	↑ IL-6 levels in blood after exposure to laboratory-evoked pain (Pressure Pain Testing) in women than men	Mun et al. (2020)

CSF, cerebrospinal fluid; FLS, synovial fibroblasts; GRO-α, growth-regulated oncogene *α*, LIF, leukemia inhibitory factor, M-CSF, macrophage colony-stimulating factor; MCP-3, monocyte chemotactic protein-3; MIF, macrophage migration inhibitory factor, MIG = monokine induced by gamma interferon, SF, synovial fluid; TMJOA, temporomandibular joint osteoarthritis.

### Macrophages and the CCL2/CCR2 Axis

**FIGURE 2:**
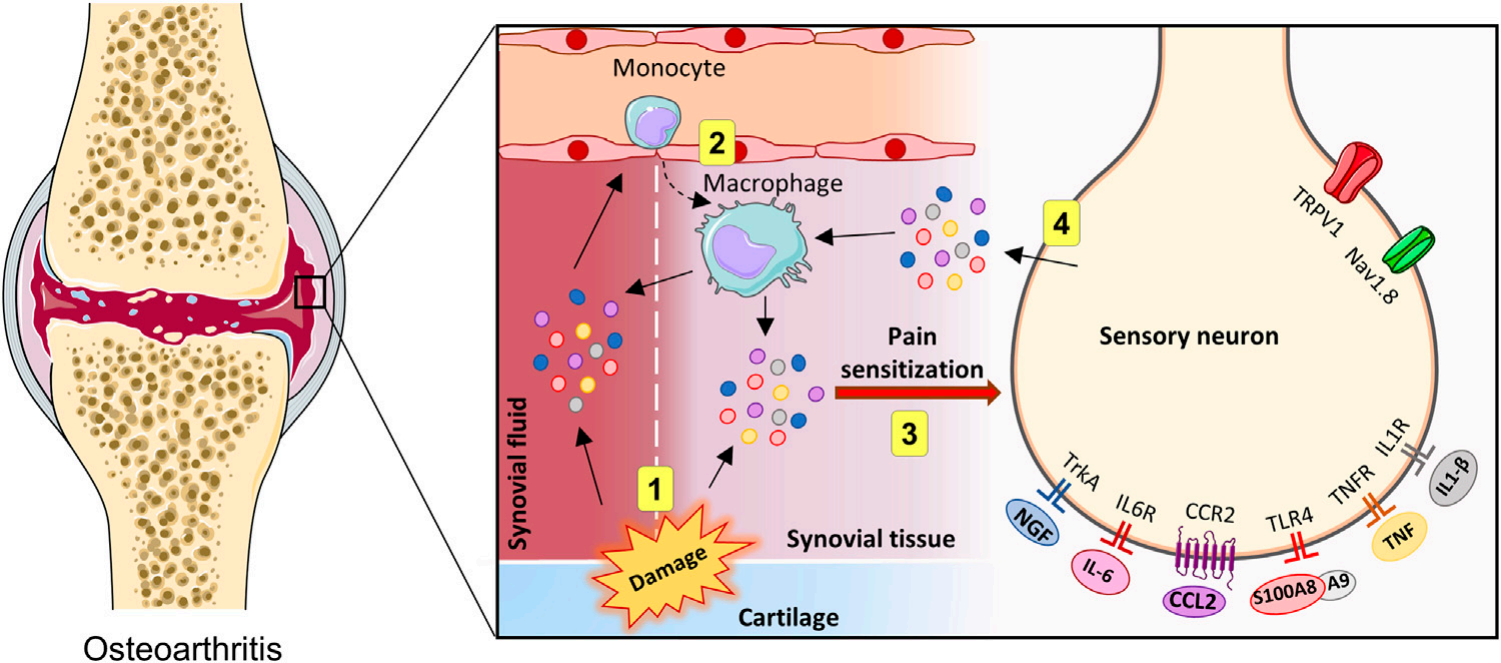
Innate immune activation in the joints and crosstalk with OA pain. Representation of direct and indirect actions of innate immune system activity on sensory neurons in the joints (1–4). During OA, tissue injuries accumulated over time may lead to the release of proinflammatory mediators, which may trigger a local innate immune system reaction (1). Then, leukocytes can be recruited, which subsequently produce additional proinflammatory factors (2). In the knee joint, proinflammatory molecules can directly excite sensory afferents neurons (3). Sensory neurons can be activated, resulting in the release of additional inflammatory mediators (4). Created with Smart. Servier^®^.

OA is associated with chronic, low-grade inflammation of the joints (Liu-Bryan and Terkeltaub, 2015; Goldring and Otero, 2011). Macrophages are main contributors to the pathogenesis of OA. Monocytes are recruited to the joints and infiltrate the synovial tissue. Once in the tissue, monocytes differentiate into macrophages, which produce inflammatory cytokines and chemokines that are released in the synovial tissues and fluid (Kapoor et al., 2011; Chen et al., 2020). OA severity and symptoms correlate with the number of activated macrophages in the joints of OA patients (Kraus et al., 2016). In this sense, accumulating evidence suggests that monocytes/macrophages are intrinsically associated with OA pain (Geraghty et al., 2021). This is logical as the proinflammatory mediators released by macrophages can bind to receptors expressed by sensory neurons, triggering signaling pathways that lead to their excitability and hypersensitivity to pain stimuli (Figure 2). Additionally, as mentioned previously, a bidirectional interaction occurs, as the sensory neurons, in turn, can also produce factors that trigger a response in the joint. The activation of nociceptors in sensory neurons produces a plethora of molecules that can directly act on macrophages, with the potential of switching their phenotypes and maintaining a pro-inflammatory milieu. For example, during tissue injury, alarmins such as S100A8/9 can activate the TLR4 present in sensory neurons, inducing the production and release of CCL2, a chemoattractant for monocytes and macrophages (Miller et al., 2015). Once recruited to the joint tissue, macrophages can produce and release additional proinflammatory mediators, inducing a positive feedback loop that may contribute to a proalgesic mechanism in the context of OA.

Macrophages are the most abundant immune cell type in the OA synovium. These cells produce nerve growth factor (NGF) (Caroleo et al., 2001), an important modulator of pain. Interestingly, in OA synovium, macrophages are also a notable source of NGF (Takano et al., 2017). Evidence from preclinical and clinical studies has suggested that the presence of macrophages in the joints and DRGs, direct or indirectly contributes to the generation and maintenance of pain in OA (for a review, see (Geraghty et al., 2021)). In murine models of OA, macrophage depletion has been associated with reduction of OA symptoms, including osteophyte formation (Blom et al., 2004) and pain (Raoof et al., 2021). In a clinical study, activated macrophages found in 76% of OA knee, were associated with OA pain severity and radiographic OA severity (Kraus et al., 2016). Also, in a cohort study (n = 86), macrophages were the most abundant of all leukocytes analyzed in the synovial fluid of OA patients and were correlated with knee injury and pain (Gomez-Aristizabal et al., 2019). Differences in macrophage phenotype also appears to contribute to the OA pathogenesis and studies in this direction have been conducted (Wood et al., 2019; Chou et al., 2020), although further investigation is needed. In addition to the synovium and synovial fluid, DRG macrophages have also been described to contribute to both the initiation and persistence of OA pain (Raoof et al., 2021). However, whether these mechanisms in which macrophages are involved in OA pain are different between men and women is unknown.

As mentioned above, TLR4 activation in sensory neurons results in CCL2 production (Miller et al., 2015). CCL2 is an important contributor to the pathogenesis of OA, including pain. CCL2 can bind to its receptor, chemokine receptor type 2 (CCR2), and modulate the infiltration and migration of monocytes and, to a lesser extent, T, dendritic, and natural killer cells into the inflammatory sites (Griffith et al., 2014). Local CCL2/CCR2 signaling in the joint contributed to knee hyperalgesia in experimental OA, which was mediated through direct stimulation of CCR2 expressed by intra-articular sensory afferents by CCL2 (Ishihara et al., 2021). Mice lacking CCL2, or its receptor CCR2, were protected against OA in a model of destabilization of the medial meniscus (DMM), with a reduction in the local monocyte and macrophage numbers in the joint (Raghu et al., 2017). Consistently, CCR2 and CCL2 deficiencies have been associated with reduced OA-related pain in mice (Miller et al., 2012; Miotla Zarebska et al., 2017). The effect of the CCL2/CCR2 axis on DRGs was also evaluated. DRGs showed increased CCL2/CCR2 production 8 weeks after DMM induction, which correlated with movement-provoked pain behaviors, which were maintained for up to 16 weeks. Notably, mice that lacked CCR2 displayed no macrophage infiltration into the DRGs (Miller et al., 2012).

The findings in rodents were corroborated by those in humans. Recently, CCL2 and CCL3 (involved with polymorphonuclear recruitment) levels were higher in the peripheral blood of OA patients than in healthy donors. The authors considered CCL2 and CCL3 as suitable predictive markers for the occurrence, efficacy, and prognosis of recurrence of OA after a 1-year follow-up study (Guo et al., 2021). In a recent meta-analysis, in which nine clinical studies were evaluated, including 376 patients with OA and 306 healthy controls, reinforced the finding that CCL2 may serve as a biomarker for the diagnosis of OA and may play an important role in the progression of OA (Ni et al., 2020). Importantly, in humans, CCL2 may contribute to driving peripheral sensitization in the OA joint, as another study reported that CCL2 levels in synovial fluid positively correlated with pain and physical disability in patients with OA (Li and Jiang, 2015).

The neuroimmune interface plays a critical role in the development and maintenance of chronic pain (Grace et al., 2014). Researchers evaluated the cerebrospinal fluid (CSF) of OA patients and controls, and found that the CSF of OA patients had higher CCL2 levels, indicating a neuroinflammatory response in OA patients (Kosek et al., 2018). They found no differences in CCL2 levels between men and women. However, in women OA patients, CCL2 levels were significantly higher in CSF than in serum, with significant positive correlations for CCL2 across the CSF, serum, and SF, but not in men, indicating differences in neuroimmune signaling in OA. Women patients reported higher pain ratings, generally more severe symptoms, and increased pressure pain sensitivity compared to men, but these observations did not correlate with CCL2 levels. However, the authors suggested that CCL2 is likely involved in neuroimmune bloodborne joint-to-CNS signaling in women but not men OA patients (Kosek et al., 2018). Notably, the OA synovial fluid of women displayed a higher percentage of macrophages in comparison to men with OA, but their percentage of monocytes was lower (Kriegova et al., 2018). Altogether, these preclinical and clinical findings highlight that macrophages and the CCL2/CCR2 axis play a key role in OA-associated pain, but further investigation into the differences between the sexes is needed.

### Alarmins S100A8/S100A9

The findings of preclinical studies have suggested that S100A8/S100A9 proteins are involved in the pathogenesis of OA. S100A8 and S100A9 are classified as alarmins belonging to a group of DAMPs, and can be produced by leukocytes, including neutrophils, monocytes, and activated macrophages (Wang et al., 2018). S100A8 and S100A9 are important regulators of the innate inflammatory response. These alarmins can form heterodimers that signal via TLR4 and mediate signal transduction pathways, which results in the upregulation of a wide range of proinflammatory cytokines, including IL-1β, TNF-α, and IL-6 (Goh and Midwood, 2012; Vogl et al., 2018). TLR4 is upregulated in the synovial tissue of OA patients, in the synovial membranes (Radstake et al., 2004), as well as in articular cartilage lesions (Kim et al., 2006).

In experimental OA models, increased S100A8 and S100A9 mRNA and protein levels were observed after chondrocyte stimulation with IL-1. Additionally, mRNA expression of S100A8 and S100A9 in chondrocytes increased early but not late in the DMM model (Zreiqat et al., 2010). In the collagenase-induced OA model, high and prolonged expressions of S100A8 and S100A9 in the synovium were reported (van Lent et al., 2012). Elevated expression of S100A8/A9 was observed in synovial biopsy samples from OA patients, which significantly correlated with synovial lining thickness, cellularity in the subintima, and joint destruction. Levels of S100A8/A9 serum protein were significantly enhanced (19%) at baseline in patients who had pronounced progression of joint destruction after 2 years (van Lent et al., 2012). In addition, findings have shown that S100A8/9 can mediate pain in OA. S100A8 is able to increase neuronal excitability on DRGs dependent on TLR4, which resulted in the release of CCL2 by DRGs (Miller et al., 2015). More recently, increased expression levels of neuron activation markers in the DRGs of WT mice, but not in *S100A9* ^*−/−*^ mice (Blom et al., 2020), were observed in a model of synovitis induced by streptococcal cell wall (SCW). In the same study, the authors found that *S100A9* ^*−/−*^ mice exhibited less pain behavior than WT mice in SCW synovitis. Much is still unknown about the involvement of S100A8/9 in TLR4 activation and its relationship to pain perception. Further investigations considering sex differences, particularly in OA pain, should be conducted as those so far were performed only in males.

### Mast Cells and NGF

Another cell type with increased levels in the synovial tissue of OA patients is the mast cells (Buckley et al., 1998; de Lange-Brokaar et al., 2016). Mast cells belong to the innate immune system, and promptly respond to exogenous or endogenous danger signals through the degranulation and release of mediators such as histamine, chemokines, cytokines, and proinflammatory lipids (Theoharides et al., 2012). The production and release of these proinflammatory mediators are partly dependent on the activation of TLRs (Sandig and Bulfone-Paus, 2012). A large body of evidence suggests that the activation of mast cells and the release of these inflammatory mediators affect the nervous system and have implications in different pain conditions (Mai et al., 2021). In OA, the presence of mast cells correlates with the synovitis score, as well as with increased structural damage (de Lange-Brokaar et al., 2016). Mast cells can produce NGF (Leon et al., 1994), which causes hyperalgesia (Lewin et al., 1993). NGF levels are remarkably higher in inflamed tissue and elevated in OA synovial fluid (Aloe et al., 1992). In addition to mast cells, macrophages (Takano et al., 2017), synovial fibroblasts (Stoppiello et al., 2014), and chondrocytes (Blaney Davidson et al., 2015) are responsible for producing NGF in the joints of OA patients. Some preclinical studies reported that anti-NGF antibody therapy is effective in reversing OA pain (Sousa-Valente et al., 2018; Sakurai et al., 2019). This effect was also reported in clinical studies, where anti-NGF-β therapy resulted in substantial pain reduction in OA patients, but with associated adverse side effects (Chevalier et al., 2013). Although no difference in NGF expression was reported in the DMM model, when comparing male and female mice (von Loga et al., 2020), the findings of a recent clinical study showed that women displayed a higher magnitude of NGF-induced mechanical sensitization than men (Alhilou et al., 2021). This can in part explain the increased susceptibility of women to experiencing pain in OA. In addition to this difference in the NGF response, women’s mast cells may produce and release more inflammatory mediators, such as histamine and TNF-α, than those of men (Mackey et al., 2016), which reinforces the predisposition of women to developing certain types of disease and related symptoms such as pain.

### Inflammatory cytokines

Proinflammatory cytokines have been implicated in driving OA progression and symptoms (Blom et al., 2007). The findings of several preclinical studies have shown that cytokines, including IL-6, TNF-α, and IL-1β, can increase the excitability of sensory neurons, leading to peripheral sensitization and mechanical hyperalgesia in joint diseases (Brenn et al., 2007; Binshtok et al., 2008; Richter et al., 2010; Mailhot et al., 2020). These cytokines can also regulate NGF expression in the synovium, which may contribute to OA pain (Takano et al., 2017).

The synovial fluid composition of end-stage OA patients shows that women have higher levels of inflammatory cytokines and other proinflammatory mediators than men (Pan et al., 2016; Kosek et al., 2018). Men show higher levels of catabolic enzymes (Solheim et al., 2017) and anabolic growth factors (Pan et al., 2016). The findings of a preclinical study with rat synovial membrane and synoviocytes corroborates these differences. Female rat synoviocytes responded with higher productions of iNOS, IL-1β, and CCL2 compared those of male rats when challenged with TNF-α. These cells were also capable of attracting more macrophages *in vitro* (Xue et al., 2018). The authors also reported similar changes in the synovial membrane in a temporomandibular joint OA model. Female rats exhibited higher OA severity and expression of iNOS, IL-1β, CCL2, and CD68 (a macrophage marker) (Xue et al., 2018). However, researchers in another preclinical study in a murine inflammatory OA model (CiOA) did not find any significant differences between the sexes in macrophage infiltration in the joints (Montgomery et al., 2020).

In clinical studies, researchers have also evaluated correlations between inflammatory cytokines and OA-related pain (Orita et al., 2011b; Leung et al., 2017; Radojcic et al., 2017; Ren et al., 2018; Nees et al., 2019). In most of these studies, no correlations were observed between inflammatory cytokines and pain when comparing women and men. However, a few recent studies have described some important observations. Perruccio et al. assessed the levels of the cytokines IL-6, IL-8, IL-10, IL-1β, and TNF-α in the blood samples of OA patients. TNF-α levels were higher in men than in women, although women reported higher knee pain scores than men. In this same study, the relationships between IL-1β and IL-8 and pain were positive for men and negative for women, while the relationship between IL-6 and pain was negative for men and positive for women (Perruccio et al., 2019). Consistent with this latter observation, the findings of another study showed that women patients scheduled for total knee arthroplasty displayed higher IL-6 reactivity after exposure to laboratory-evoked pain than men. This indicated that IL-6 may contribute to the maintenance and/or exacerbation of OA pain in women (Mun et al., 2020). In another study, women patients reported more severe pain and had higher IL-8 levels in SF and more IL-8 mRNA expression in cartilage than men patients. Additionally, a positive association was found between IL-8 in SF and pain scores in women but not men patients, indicating the different effects of IL-8 in SF depending on sex (Kosek et al., 2018). Regarding the levels of IL-6, SF concentrations of IL-6 were higher than those in the CSF and serum in both sexes. However, no significant sex differences between correlation coefficients were found in that study. Altogether, these results have shown the contribution of inflammatory cytokines to OA pain, but their specific roles particularly when considering sex remain unclear, so further studies should be conducted.

**FIGURE 3:**
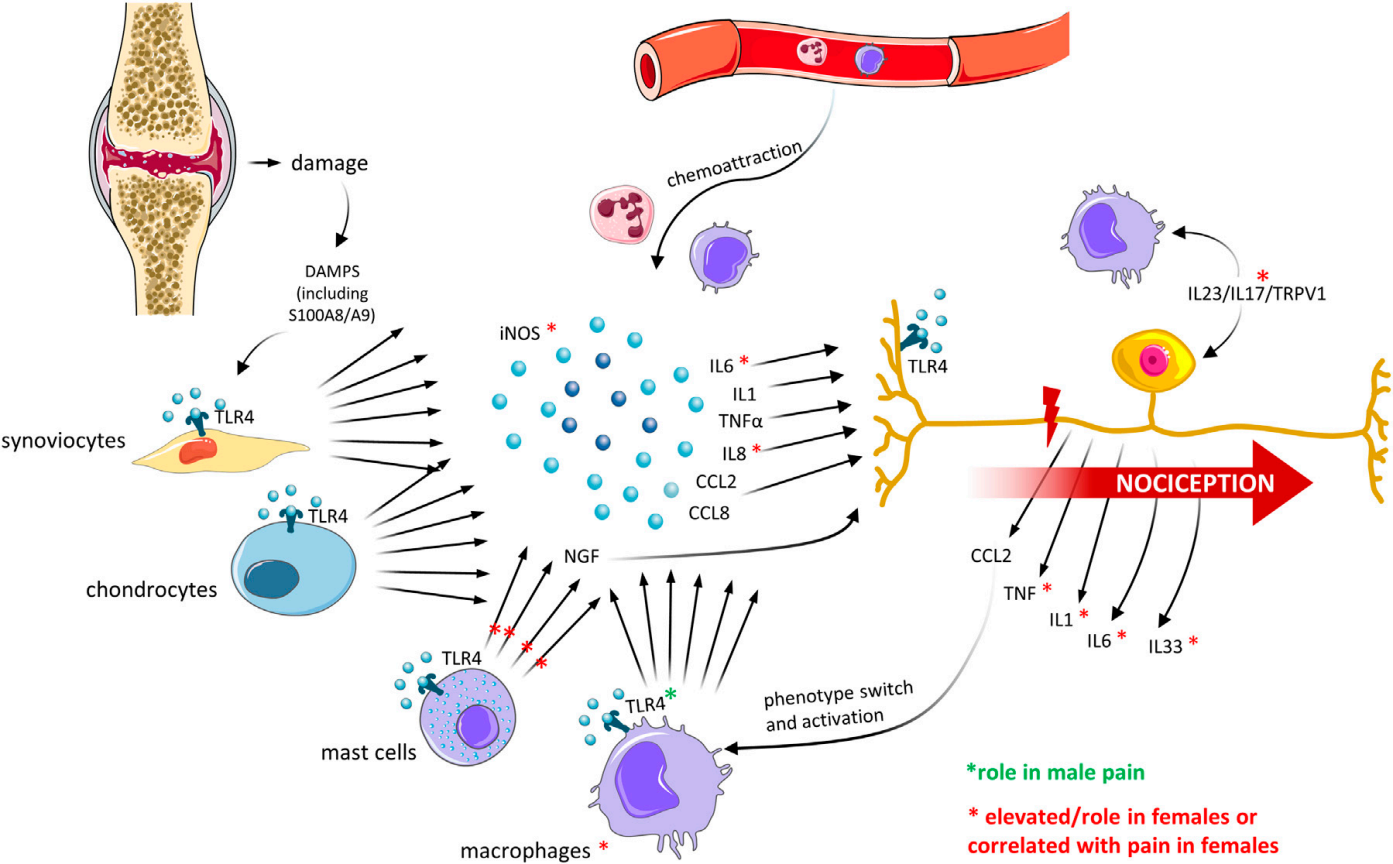
Sex differences in innate immune mediators in the joint and crosstalk with OA pain. Recent OA preclinical and clinical studies have described differences in the expression of mediators of the innate immune system, which correlated with differences in pain perception between men and women. The major findings are described. Created with Smart. Servier^®^.

So far, researchers have focused on possible sex differences in the innate immunity in the joint compartment, indicating the nociceptive trigger might differ between sexes (Figure 3). However, whether the differences are only quantitative or qualitative and the biological mechanisms behind these differences remain unclear.

## Sex Differences in the Pain Compartment

Regardless of whether the nociceptive input in the joint is sexually dimorphic, recent evidence suggests that primary afferent machinery transmitting the pain signal to the CNS may differently process the signal between sexes. Two biologically distinct pain pathways between sexes in preclinical studies have recently gained increased scientific interest, and they are intimately linked to the innate immunity.

Pain hypersensitivity after nerve injury or inflammation is dependent on spinal TLR4, which is linked to the microglia (macrophage-like cells residing in the central nervous system tissue) in male but not in female mice. This pain mechanism is testosterone- and site-dependent (Sorge et al., 2011; Sorge et al., 2015). Despite the initial TLR4-dependence in male pain being suggested to be in the spinal compartment, intrathecal injections were performed, which also include the DRG (Wang et al., 2005) (spinal) TLR4 has also been demonstrated to mediate the transition to a persistent mechanical hypersensitivity after the resolution of inflammation in serum-transferred arthritis in male mice. However, female mice were not included in this study (Christianson et al., 2011). With (spinal) TLR4 signaling possibly explaining sex differences, many groups have been investigating its role in inflammatory pain, the development of persistent pain states, and sex differences in pain. In an experimental model of arthritis, the blockage of the peripheral TLR4 ligand, HMGB1, reversed hypersensitivity in male mice, but had little effect in female mice. The authors also showed that this sex-dimorphic response to TLR4 was dependent on macrophages and microglia; removal of TLR4 from sensory neurons did not result in hypersensitivity in male or female mice (Rudjito et al., 2021). Other findings by the same group also supported this finding: TLR4 in the sensory neurons is required for female mechanical hypersensitivity upon nerve injury (Szabo-Pardi et al., 2021). Hence, TLR4 seems to be important for pain in both sexes (in the sensory neuron), but is sexually dimorphic in the macrophages and microglia. Findings of an investigation into the possible mechanisms of the sex-dependent role of microglia and TLR4 activity showed that intrathecal injection of HMGB1 resulted in an equal increase in microglial immunoreactivity in the spinal cord. However, when investigating microglia response to TLR4 stimulation *in vitro*, male microglia responded with higher cytokine and chemokine expressions than female microglia (Agalave et al., 2021).

The hypothesis that females do not require the (spinal) microglia for pain processing has led to multiple efforts toward elucidating the female-specific mechanism. Females might use T cells from the adaptive immune system to produce chronic pain hypersensitivity (Sorge et al., 2015). However, the role of T-cell infiltrating in the spinal cord has been debated given contradictory findings (Gattlen et al., 2016). Recently, more light was shed on the female pain pathway; again, the neuroimmune interaction focusing on innate immunity was highlighted. Mechanical pain in females is mediated through macrophage–neuron crosstalk via the IL23/IL17/TRPV1 axis in the DRG. Sex dimorphism was shown at the immune level, with females macrophages producing more IL23 and IL17A, and at the neuronal level, with female nociceptors being more sensitive to IL17A. The findings also showed that this mechanism is dependent on estrogen and is independent of T cells (Luo et al., 2021). The lack of T-cell involvement in female’s pain indicates the innate immune system (macrophages) as the core of sex differences in pain, and provides further motivation for additional research.

These preclinical findings on sex differences in pain pathways have created a new scientific field with high potential for new pain target discoveries. The role of innate immunity in the DRG in pain processing in general is also a promising new area of research (previously, pain was thought to only involve neurons). Transcriptomics data mapping sex differences in naïve DRG demonstrated that female murine sensory neurons expressed higher levels innate-immunity-related genes, such as chemokines, cytokines (IL1, IL6, and IL33), and the tumor necrosis factor superfamily (Mecklenburg et al., 2020). This study also showed that male murine naïve sensory neurons had higher gene expression levels related to anabolism, including protein metabolism. These differences may explain the tendency for female sensory neurons to shift the balance between neuroimmunity and neuroimmunopathology, more easily promoting the transition from acute to chronic pain. Conversely, male sensory neurons have machinery capable of more efficient repair and resolution (Mecklenburg et al., 2020). However, these are gene expression indications, which are influenced by the translation machinery, so they finally regulated in nociception pathways and are involved in the development of pain conditions.

Researchers investigating transcriptomics in human DRG from neuropathic pain patients also found sex differences. Despite a smaller cohort, differential gene expressions in a human-specific macrophage lineage were observed between pain cohorts of men and women. In men, the upregulated genes included CXCL2, TNF, and several transcription factors of the FOS-JUN family. In women, upregulated genes included several class A rhodopsin-like G-protein coupled receptors (CX3CR1, ADORA3, P2RY13, and GPR65). Altogether, these findings suggested that some of the sex-differential gene expression in (neuropathic) pain samples may also be driven by macrophages in humans (North et al., 2019).

In OA, DRG microarray analyses in the DMM model also support the role of innate neuroimmune pathways in the initiation and establishment of persistent pain. Initially, genes related to the movement and activation of immune cells start to increase in the early OA pain phase, transitioning to increased gene expressions related to immune cell recruitment, proliferation, and activation in the persistent OA pain stage. In this later stage, CX3CL1, CCL2, TLR1, and NGF are upregulated (Miller R. E. et al., 2020). This study was performed using male mice, so sex differences could not be studied. In a recent study using the monoiodoacetate-induced OA model, the authors found that DRG macrophages play a primary role in the maintenance of OA pain (Raoof et al., 2021). The authors identified that DRGs were infiltrated with macrophages shaped with an M1-like phenotype. These macrophages were responsible for remotely maintaining OA pain, independent of joint damage. Notably, depletion of macrophages or switching the phenotype of DRG macrophages to the M2-like profile resolved OA pain. In this later study, both male and female mice were included in the pain measurement experiments and no differences between the sexes were observed. Depletion of macrophages from the DRG, however, effectively reduced mechanical hypersensitivity in both sexes (Yu et al., 2020). To the best of our knowledge, this is the first OA study that demonstrated a role of macrophages in OA pain as well as possible sex differences in the DRG. Nevertheless, the gaps in knowledge of the role of innate immunity and sex differences in OA pain require further study.

## Conclusion

Since the early 2010s, sex-inclusive research has been gaining relevance and has been amplified by decision makers within government funding bodies. These major changes have been implemented as governments became aware of a widespread sex bias in research and its impact on healthcare. In pain, the high failure rate of new analgesic drugs in clinical trials after successfully passing preclinical investigation was notable. These failures were particularly high in pain conditions with a higher incidence in women (Berge, 2011). These issues arose because male animals were the preferential choice for preclinical and clinical studies, which was mainly based on the concept that hormone fluctuation in female animals increases experimental variability, a concept that has been disproven as male subjects show more variability than female subjects in experimental settings. Added to that was the notion that no major differences between sexes exist outside reproductive functions; therefore, the idea was that what was discovered for one sex would apply to the other. In clinical and epidemiological research, sex was also overlooked as a potential biological variable. If and when both sexes were included, data were analyzed correcting for sex, which could have hidden possible sex differences.

**FIGURE 4:**
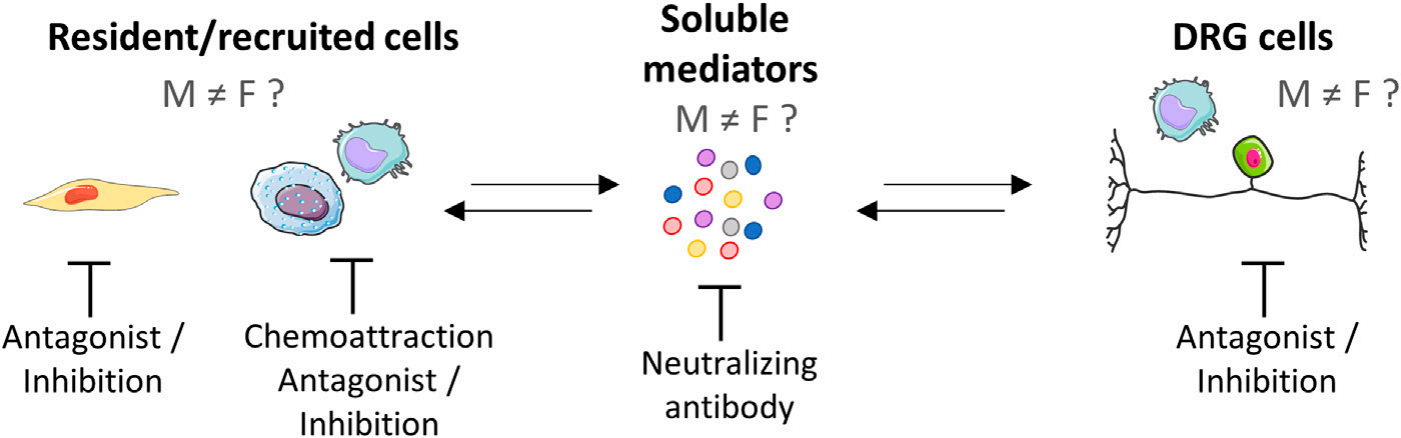
Peripheral neuroimmune interaction and potential pharmacological targets. The neuroimmune interaction is a bidirectional process that can be mediated by soluble factors that drive a complex crosstalk among nerves and immune cells. Studies evaluating sex differences in this context are emerging and have found differences between the sexes. Potential pharmacological targets are represented, e.g., resident cells (synovial fibroblasts and macrophages), recruited immune cells (macrophages and mast cells), soluble mediators, and neurons. As differences in neuroimmune interaction have already been described, differences in the therapeutic approach between men and women should be addressed in future research. Created with Smart. Servier^®^.

Excluding women from the initial stages of drug trials has led to a lack of data on how drugs affect them. Men and women respond differently to analgesics (Packiasabapathy and Sadhasivam, 2018). Recently, preclinical studies have shown that males and females have biologically distinct pain pathways, especially considering the neuroimmune interactions. However, data on the impact of such differences in the context of OA pain are still scarce. In this review, we described preclinical and clinical studies in which researchers examined the role of the immune system in OA pain in consideration of sex differences. The existing information provides evidence of the immune-related pathophysiology of OA as well as promising avenues for OA research and treatment. We described the close interaction between OA pain and innate immunity mediators, with a focus on sex differences. Despite the efforts in this field, a large gap remains to be filled as, until now, most research has been preferentially conducted in male subjects. When both sexes started to be included in preclinical and clinical studies, the differences between sexes became more apparent. Thus, elucidating these sex-dependent differences within the molecular pain pathways will provide grounds for the development of novel therapies with a focus on specify targets considering a sex-based approach (Figure 4). Given the vast interaction between innate immunity and pain, elucidating which factors are dominant in pain, in addition to differences between men and women, is still a large scientific gap to fill.

## Glossary

### Pain types - designated according to the nature of the pain stimulus

Nociceptive: activation from high threshold sensory neurons (nociceptors);
Inflammatory: resulting from persistent inflammation;
Neuropathic: involves direct or indirect primary afferent nerve or ganglia damage.

### Pain sensations denominations can vary according to the stimulus

Allodynia: pain provoked by an innocuous stimulus;
Hyperalgesia: increased pain perception from a painful stimulus.

### Other pain terms

Summation: process of adding up the effect of multiple subthreshold stimuli, that determine whether or not an action potential will be generated by combined effects.
Spatial summation: effect of triggering an action potential from multiple simultaneous inputs;
Temporal summation: effect of triggering an action potential from repeated inputs.
Other terms: Male/female, men/women: Sex and gender are commonly used as interchangeable terms in medicine, however, they are two different concepts. Sex refers to sets of biological traits of people and animals and usually is categorized as male and female (and intersex), while gender refers to a more complex interaction between socially constructed roles, behaviors, expressions and is not binary. Here, we used the terms male/female for studies in animals and, men/women patients for studies in humans.
